# Negative Fat Pad Biopsy in Systemic AL: A Case Report Analyzing the Preferred Amyloidosis Screening Test

**DOI:** 10.3390/diseases9020040

**Published:** 2021-05-28

**Authors:** Kelsey Hummel, Hany Meawad, William Gunning, Amira Gohara

**Affiliations:** 1 Department of Pathology and Immunology, Baylor College of Medicine, Houston, TX 77030, USA; 2 City of Hope Laboratories, Duarte, CA 91010, USA; hameawad@coh.org; 3 Department of Pathology, University of Toledo Medical Center, Toledo, OH 43614, USA; William.Gunning@utoledo.edu (W.G.); Amira.Gohara@utoledo.edu (A.G.)

**Keywords:** amyloidosis, biopsy, negative, rectal, fat pad, bone marrow, autopsy, AL

## Abstract

Light chain amyloidosis (AL) causes irreversible multi-organ damage if not diagnosed early in the disease process. Fat pad biopsy is thought to be a highly sensitive screening test in systemic AL cases, especially if greater than three organs are involved. We present a case of a 64-year-old female who was admitted to the hospital with worsening heart and kidney failure, anasarca, increased free serum lambda light chains, and a negative fat pad biopsy for amyloidosis. Later, she developed asystole, bradycardia, severe hypotension, and respiratory distress. Because X-rays of her calvarium showed multiple osteolytic lesions, a bone marrow biopsy was planned to assess for multiple myeloma. Due to her non-reassuring vitals, the biopsy was not attempted, and she passed away several weeks later. Autopsy findings identified the cause of death as multiple system organ failure due to systemic AL. Through microscopic examination, pathologists found amyloid deposits in her heart, kidneys, rectum, thyroid, adrenals, bone marrow, liver, and spleen. Postmortem fat pad biopsy was negative; however, bone marrow biopsy demonstrated clusters of CD138-positive cells, confirming plasma cell dyscrasia. In cases with a negative fat pad biopsy, an additional superficial or involved organ biopsy should be pursued to establish a diagnosis of amyloidosis if strong clinical suspicion exists.

## 1. Introduction

Amyloidosis is a disease of abnormal protein accumulation in organ tissues. Light chain amyloidosis (AL), the most common form, is often caused by plasma cell dyscrasia, such as multiple myeloma [1]. In contrast, transthyretin (ATTR) and autoimmune (AA) subtypes occur because of an unstable protein produced in several organs (i.e., liver, choroid plexus, and retina) or chronic inflammation that leads to increased amyloid deposits [2,3,4]. For patients with unexplained nephrotic-range proteinuria and concurrent heart disease, physicians are recommended to assess for amyloidosis. To initiate appropriate therapy and prevent irreversible organ failure, timely diagnosis of amyloidosis and its cause is crucial [5,6].

The fat pad biopsy is inexpensive, sensitive, non-invasive, and the preferred screening test for amyloidosis [7]. However, studies indicate the sensitivity of this test depends on several factors, including extent of organ involvement, operator skill, and amount of fat tissue submitted for microscopic examination. This variable sensitivity often leads to false negatives that delay diagnosis and result in worse outcomes for patients [6,8]. We report an autopsy case showing systemic AL causing multi-organ failure with several negative fat pad biopsies.

## 2. Case Presentation

### 2.1. Clinical Presentation

A 64-year-old Caucasian female arrived by ambulance to our emergency room complaining of difficulty breathing. She was managed at several other hospitals before, but stated that her symptoms steadily became worse. She presented with generalized weakness, anasarca, nephrotic syndrome, decompensated heart failure, and leukocytosis with neutrophilia. Her serum protein electrophoresis showed hypoalbuminemia with hypo-gammaglobulinemia, as well as increased lambda and kappa light chains. Additionally, her urine tested positive for lambda light chains with nonselective uropathy. Her chest X-ray demonstrated cardiomegaly and bilateral pulmonary opacities. When our physicians discovered increased gamma-glutamyl transferase (gamma-GT) antibody levels, they suspected amyloidosis. They ordered a core needle fat pad biopsy, but it was negative.

**Figure 1:**
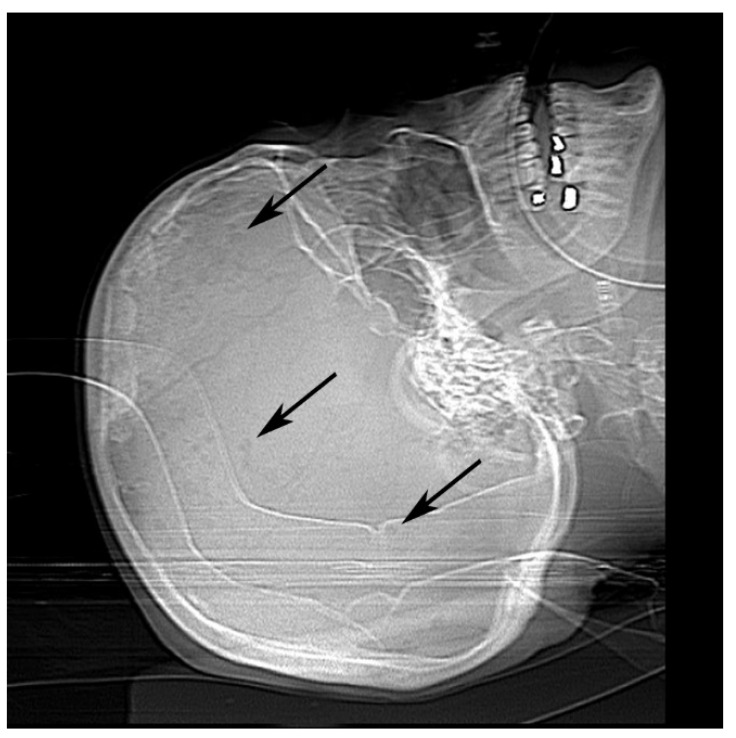
Antemortem X-ray of calvarium showed multiple punctate lucencies (black arrows).

Once physicians stabilized her heart and kidney failure, she was prepped for dialysis and discharged. She returned four days later to our emergency room with asystole. Computed tomography scan and X-ray imaging revealed chronic microvascular ischemic change with lucencies in the calvarium (Figure 1). Radiologists suggested these findings could be seen in multiple myeloma or metastatic disease.

Shortly after completing these tests, she went into respiratory failure and was intubated, then admitted to the hospital. During her stay, she developed severe hypotension (84/55 mmHg), bradycardia (20–30 beats per minute), and bilateral pleural effusion. Chest compressions were performed on multiple occasions to keep her cardiac rhythm stable, while chest tubes were frequently draining fluid to manage the effusion. To assess for multiple myeloma, her physicians scheduled a bone marrow biopsy. However, they ultimately canceled the biopsy after they deemed she was too unstable to undergo the procedure. By this time, she had been intubated in the hospital for several weeks, and was beginning to show signs of multi-organ failure in the liver (macrocytic anemia and thrombocytopenia with increased ammonia, liver enzymes, prothrombin time, and partial thromboplastin time), as well as the kidney (burr cells and schistocytes on peripheral smear). Her family decided to withdraw care and consented for a full autopsy without any limitations performed by the hospital.

### 2.2. Amyloid Detection

#### 2.2.1. Antemortem Biopsy

Clinicians performed a bed side core needle fat pad biopsy, in which three cores measuring 20 mm in length by 2 mm in diameter each (62.8 mm^3^ total) were submitted. Pathologists examined the biopsies using light microscopy with hematoxylin and eosin (H&E) and Congo-Red stained slides, as well as electron microscopy.

#### 2.2.2. Postmortem Biopsy

With a scalpel, a pathologist removed a thin piece of subcutaneous fat measuring 20 × 3.5 × 3 mm (210 mm^3^) from the area peripheral to the umbilicus. A second pathologist obtained a larger excisional fat pad biopsy from the area opposite of the first postmortem biopsy. The second biopsy was obtained using the procedure described by Garcia et al. and measured 9 × 8 × 8 mm (576 mm^3^) [9]. H&E and Congo-Red stains were utilized during light-microscopic examination of the biopsies. Fat pad, spleen, kidney, liver, and rectum samples were obtained for electron microscopy. Through immunofluorescence microscopy, pathologists inspected additional kidney tissue for lambda and kappa light chains. Our laboratory was unable to process the specimen for ATTR or further subtypes.

#### 2.2.3. Light Microscopy with Polarized Filter

Received tissue samples were fixed for several hours in a 10% buffered formalin solution, then processed overnight (Leica Biosystems, Wetzlar, Germany). The processed tissue was embedded in paraffin wax and a 4 µm thick section was mounted on a charged glass slide. Then the sections were stained with H&E and Congo-Red. Pathologists examined the H&E slides via routine light microscopy. At the same microscope, they examined the Congo-Red slides using a detached polarizing filter.

#### 2.2.4. Immunofluorescence Microscopy

Our laboratory technologists applied a direct-labeling immunofluorescence protocol to kidney samples by using goat anti-human, fluorescein-conjugated antibodies for lambda and kappa light chains (Vector Laboratories, Inc., Burlingame, CA, USA). Sections obtained from the paraffin block were mounted on charged glass slides. After deparaffinizing the sections in xylene and hydrating with distilled water, slides were placed in a hot citrate solution for antigen retrieval. Subsequently, the sections were bathed in nonimmune horse serum, which acted as a blocking agent. Each slide consisted of two sections—a section for an antibody reaction and a negative control section where no antibody was applied. After a thirty-minute incubation followed by a buffer wash, a cover slip was mounted with glycerin and examined by an immunofluorescence microscope.

#### 2.2.5. Electron Microscopy

For the transmission electron microscopy (TEM) analysis, tissue samples were fixed with 3% glutaraldehyde buffered by 0.2 M sodium cacodylate (pH = 7.2) for thirty minutes at 37 °C. The samples were post-fixed for two hours at room temperature with 1% osmium tetroxide followed by one hour in a saturated, uranyl-acetate aqueous solution for tertiary fixation. The tissue on the slides were then dehydrated through a graded series of ethanol. The culture plates were scraped with a rubber policeman and mixed with 100% acetone with the embedding media. Then, tissue samples were embedded in Embed 812 epoxy resin (Electron Microscope Sciences, Fort Washington, PA, USA) and ultrathin sections were collected on 300-mesh copper support grids. After staining sections with uranyl acetate and Reynold’s lead citrate, the sections were examined with a FEI Tecnai T20 TEM at 80 kV. To determine the size of the amyloid fibrils, 50–80 random widths and diameters of fibrils were assessed with a calibrated measurement tool included in the Olympus Soft Imaging Radius software.

### 2.3. Autopsy Examination

#### 2.3.1. Gross Findings

**Figure 2:**
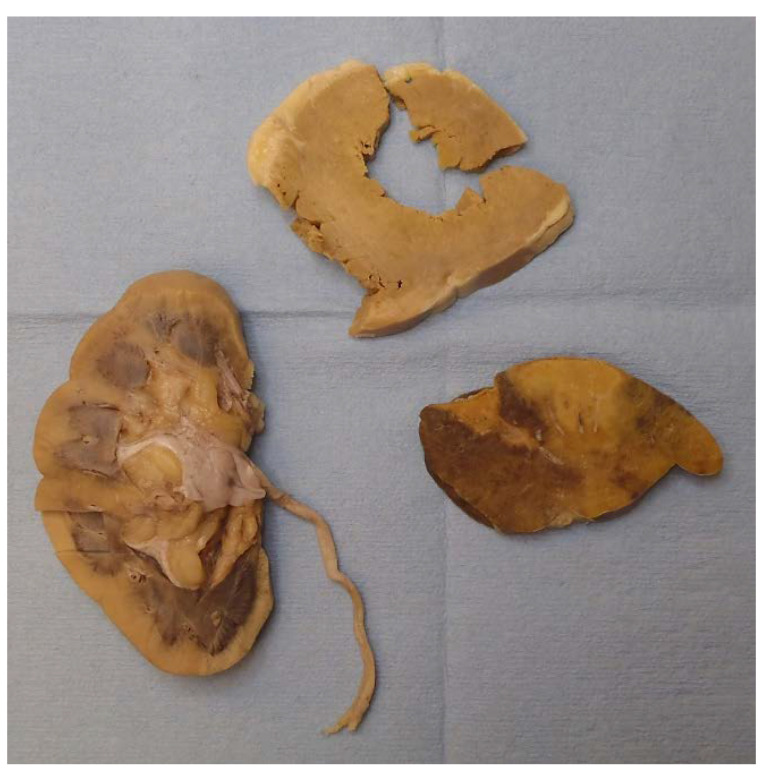
Starting in the bottom left and moving clockwise, the cortex of the right kidney, left ventricle of the heart, and spleen (lardaceous pattern) showed gross pale-yellow tissue, indicating amyloid deposit.

Gross examination found multiple organs to be firm and waxy, including the heart, spleen, kidneys, and liver. The spleen demonstrated a diffuse pale-yellow infiltrate throughout the parenchyma consistent with a lardaceous pattern (Figure 2). Sago and lardaceous are two specific gross patterns identified in spleens that are involved by amyloidosis [10,11].

The heart had right and left ventricular hypertrophy, and the cortexes of both kidneys were expanded. The thyroid, both adrenal glands, and the rectum appeared grossly normal.

#### 2.3.2. Microscopic Examination

**Figure 3:**
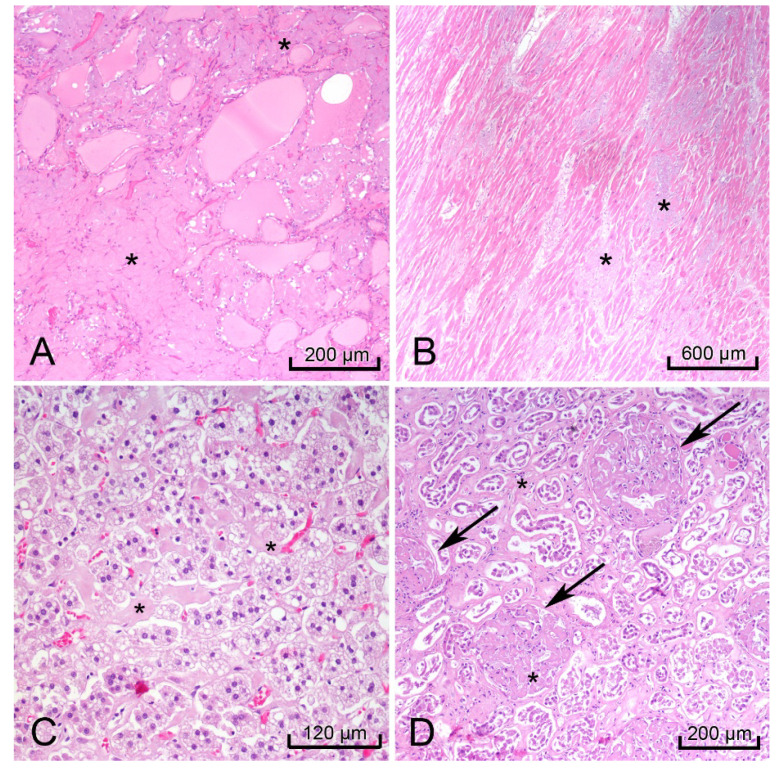
Images of hematoxylin & eosin (H&E) stained organs with hyaline extracellular matrix (black *) that indicated amyloidosis. Thyroid (**A**), heart, left ventricle (**B**), right adrenal gland (**C**), and right kidney (black arrows glomeruli) (**D**).

**Figure 4:**
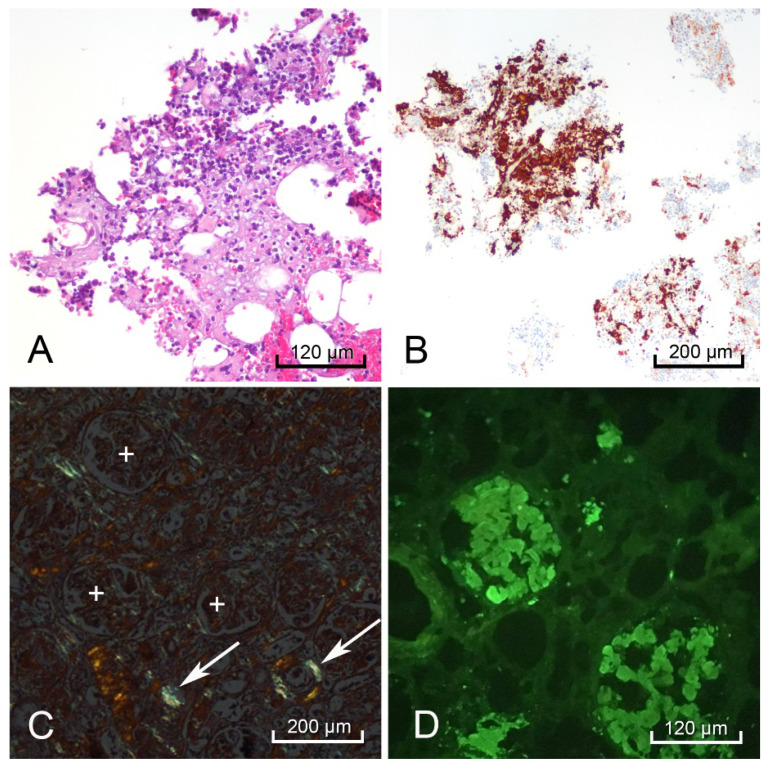
Postmortem bone marrow scraping with hematoxylin & eosin (H&E) staining showed hyaline extracellular matrix surrounding multiple cells (**A**). CD138-positive cells stained brown indicating the bone marrow contained numerous plasma cells (**B**). The right kidney demonstrated positive apple-green birefringence with Congo-Red stain in the blood vessel walls (white arrows), but not in the glomeruli (white +) (**C**), and a positive pattern with lambda light chain immunofluorescence in the glomeruli (**D**).

H&E-stained sections of the heart, thyroid, liver, spleen, rectum, kidneys, and adrenal glands showed hyaline extracellular matrix (Figure 3). In the spleen, this matrix was located within the red pulp and sinusoids, consistent with the lardaceous pattern identified on gross examination [10,11]. Bone marrow scraping of the rib revealed extracellular matrix (Figure 4A) surrounding cells with CD138-positive staining, which confirmed plasma cell dyscrasia (Figure 4B). Congo-Red stained sections of the right kidney demonstrated apple-green birefringence in blood vessel walls (Figure 4C). Immunofluorescence microscopy showed positive reactions to lambda light chains in the mesangium and basement membranes in the glomeruli of the right kidney (Figure 4D).

**Figure 5:**
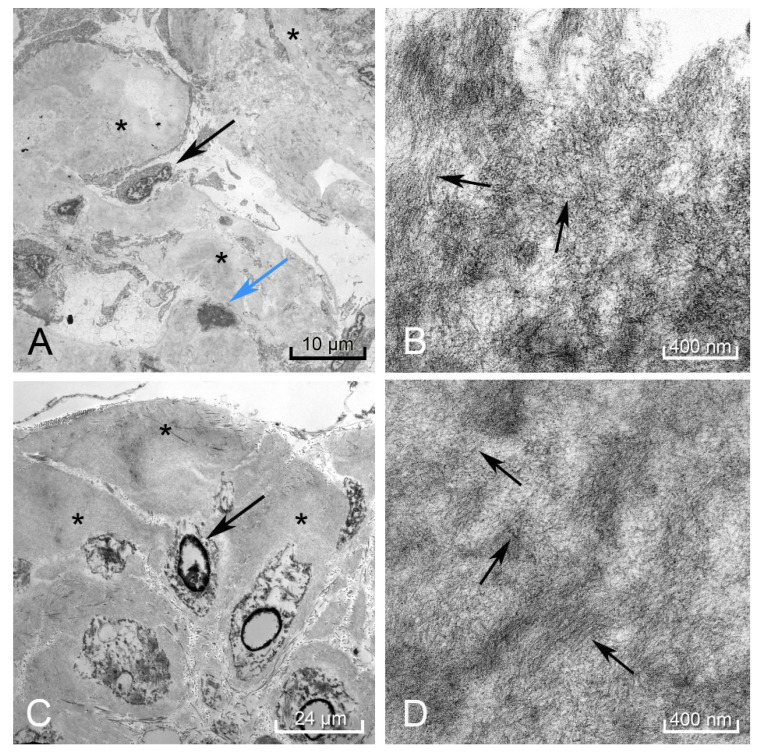
Electron microscopy of the kidney showed amorphous deposits within the mesangium and membranes of the capillary loops in the glomeruli (black arrow podocyte, blue arrow mesangial cell, black * amyloid deposit) (**A**). Higher magnification revealed these to be amyloid fibrils arranged in a haphazard pattern (black arrows perpendicular to fibril direction) (**B**). The rectum showed these same amyloid deposits (black *) surrounding the smooth muscle myocytes (black arrow) (**C**), demonstrating a similar pattern to the fibrils found in the kidney (black arrows perpendicular to fibril direction) (**D**). Autolytic changes were appreciated in the disintegrating nuclei of the mesangial cell and rectal myocytes.

In the spleen, liver, kidney (Figure 5A,B), and rectum (Figure 5C,D), electron microscopy revealed the hyalinized material consisted of 8–11 nm diameter fibrils deposited in a haphazard pattern of beaded filaments. The resolution of these fibrils was partially compromised due to the tissue being taken at autopsy instead of a live tissue biopsy.

Consistent with the negative fat pad biopsy obtained antemortem, the postmortem fat pad biopsies did not show any of these positive features on light microscopy or electron microscopy.

#### 2.3.3. Final Autopsy Report

Pathologists determined the cause of death was multi-organ failure due to systemic light chain gammopathy. Due to the presence of CD138-positive cells in the bone marrow scraping, plasma cell dyscrasia most likely caused this gammopathy. Although the patient presented with proteinuria, increased free serum light chains, and osteolytic bone lesions, clinicians did not report a diagnosis of multiple myeloma or monoclonal gammopathy of undetermined significance (MGUS) at the time of autopsy. Therefore, the final autopsy report did not include these diagnoses.

## 3. Discussion

Amyloidosis is difficult to diagnose early in the disease course due to its slow progression, non-specific symptoms (e.g., shortness of breath, fatigue, hand numbness), and low frequency of observation (i.e., 8–12 cases per million per year in the United States) [12]. Patients tend to dismiss these symptoms as signs of aging and may not discuss them with their doctors until organ damage is irreversible [9]. For some patients, a single abnormal lab test, such as protein in the urine, is the first indication there is an underlying disease process [12]. For this reason, physicians should perform a thorough workup to rule out amyloidosis in patients presenting with unexplainable nephrotic syndrome and concurrent heart failure.

A tissue biopsy is necessary to diagnose amyloidosis. A positive biopsy indicates that other diseases, such as scleroderma, may not be the cause of vague systemic symptoms (i.e., intestinal pseudo-obstruction and peripheral neuropathy) [13]. Involved organ biopsies of the liver, kidney, or heart are the gold standard to diagnose amyloidosis with 100% sensitivity and specificity [5]. Due to the tissue size and amount of amyloid deposits, involved organ biopsies enable subtype studies. These studies significantly influence treatment of amyloidosis [13]. However, involved organ biopsy has clinical limitations [13]. Amyloid proteins can deposit in vessel walls, causing the walls to become fragile and increase risk of hemorrhage during a biopsy. This translates to decreased utilization of involved organ biopsies except when performed at a specialized facility [14]. Consequently, these biopsies are obtained later in the disease course when treatments, such as chemotherapy for AL, may not prevent the patient from requiring an organ transplant [6].

This has spurred physicians to investigate procedures and tissue sites that can provide adequate diagnostic material with fewer complications. These alternate sites, known as superficial biopsies, include the rectum, gingiva, bone marrow, and abdominal fat pad. Rectal biopsies were standard practice before 1973, but due to lower expense, ability to perform in an outpatient setting, and similar sensitivity and specificity, bedside fat pad biopsies have replaced them [15,16,17,18].

The method of performing these biopsies varies across institutions. Fine needle aspiration (FNA) is the most common method as it provides diagnostic tissue and low morbidity to the patient [19]. However, this method’s sensitivity ranges from 57–85% for systemic AL and does not reliably yield enough tissue to perform subtyping studies, which are an essential component to determine appropriate treatment and disease prognosis [5,6,16,20,21]. Core needle biopsies collect more diagnostic tissue for microscopic examination, but this method also provides limited material for subtyping [9]. Using a scalpel, excisional biopsies remove a piece of subcutaneous fat that weighs approximately 1 g. This ensures a greater amount of tissue is sampled compared to the 30 mg submitted for FNA [9,21,22]. In AL cases, Garcia et al. demonstrated excisional biopsies acquire sufficient tissue for subtyping and are 100% sensitive for samples greater than 700 mm^3^ (9 × 9 × 9 mm) and 50% sensitive for those less than 700 mm^3^ [9]. Under evaluation is the punch biopsy method that includes skin with the subcutaneous tissue in order to sample the blood vessels in the dermis. As indicated by Li et al., this method increases the biopsy sensitivity as the dermis vessels may contain amyloid deposits instead of the subcutaneous fat vessels [15]. Guidelli et al.’s study found all punch biopsies performed for amyloidosis were adequate for evaluation compared to the 78% of fat pad FNAs, possibly providing another option for clinicians to utilize a simple outpatient procedure [23]. Ultimately, the procedure type used is influenced by the institution and the resources available to analyze the specimen.

Several factors can influence fat pad biopsy sensitivity. Multiple studies recommend subcutaneous fat pad biopsies and FNAs should contain at least fifteen blood vessels for adequate amyloidosis evaluation [19]. Garcia et al. reported 79% and 12% overall sensitivity on excisional biopsies for systemic disease caused by AL and ATTR subtypes respectively [9]. Compared to ATTR, AL tended to be positive more often in other superficial biopsies, such as rectum and bone marrow [8,14]. Quarta et al. revealed AL patients with low, moderate, and heavy disease burden (as determined by I-serum amyloid P scintigraphy) had fat pad FNA sensitivities ranging from 78%, 98%, and 100% respectively [14]. Van Gameren et al. reported fat pad FNAs were more likely to be positive and demonstrate a higher amyloid grade when more organs were involved [20]. This grade refers to the percentage of slide tissue involved with amyloid visualized by Congo-Red stain [20]. Additionally, Shidham et al. indicated early or localized disease tended to have less sensitive fat pad FNAs [21]. Operator expertise and initial biopsy processing at bedside can also play a role in biopsy sensitivity [19].

While a Congo-Red stain with apple-green birefringence is amyloid specific, its sensitivity varies. False negatives can occur when the stain does not react with the specimen appropriately [15]. Alternatively, inexperienced eyes can mistake collagen’s blue birefringence for amyloid deposits leading to false positives [24]. Devata et al. showed the significance of interpersonal variability with pathologists interpreting Congo-Red stains, supporting the use of ultrastructure examination [24]. Even when a Congo-Red stain is negative, electron microscopy can increase biopsy or FNA sensitivity by visualizing the 8–12 nm straight fibrils arranged in a criss-cross, unbranching formation [24,25]. Additionally, immunoelectron microscopy can be utilized to detect lambda and kappa light chains and assist with identifying the disease subtype [6]. Of note, the fatty component of a fat pad biopsies and FNAs can affect ultrastructure evaluation methods and proteomic subtyping analysis when an abundance of fatty tissue in the minute sample size leaves little to no fibrovascular cores present for adequate assessment [19]. Despite reported increased sensitivity, these ultrastructure techniques have not been adopted as the standard practice for diagnosis as many institutions do not have the equipment or expertise for these methods.

This case illustrates a unique presentation of AL: heavy disease burden involving seven organs and three negative fat pad biopsies (one antemortem core needle and two postmortem excisional). H&E staining revealed amyloid deposition in the internal organs (adrenals, kidneys, heart, liver, spleen, thyroid, rectum). These lesions were confirmed as amyloid in the rectum, spleen, liver, and kidney via electron microscopy. Congo-Red staining and polarized light also confirmed amyloid deposits with apple-green birefringence in the kidney. According to van Gameren et al.’s findings, a patient with three or more organs involved should present with at least low-grade amyloid deposits (<1% of Congo-Red stained positive lesions) on a fat pad FNA [20]. However, our postmortem excisional biopsy tested negative. This biopsy measured 9 × 8 × 8 mm (576 mm^3^), which would yield ~50% sensitivity according to Garcia et al.’s findings [9]. In their study, Garcia et al. stated the size of the biopsy makes the greatest difference in identifying these deposits in the AL subtype when the serum free light chain ratio is <0.5 or ≥5 [9]. In this case, the ratio was 0.08. Thus, we could not rule out that a greater amount of tissue was needed in order to detect low-grade amyloidosis [14,21].

Other studies indicate fat pad biopsies or FNAs can be negative when alternate superficial sites are positive [15]. This case demonstrated a positive rectal and bone-marrow biopsy via electron microscopy and clusters of CD138-positive stained cells respectively. Additionally, studies suggest that two negative superficial biopsies render the diagnosis of amyloidosis highly unlikely [15], although for some cases, involved organ biopsy may be required for diagnosis [5,6,7,8]. Therefore, additional biopsies should be pursued if the fat pad sampling is negative when clinical symptoms and laboratory results strongly suggest amyloidosis.

In this case, the physicians intended to obtain another superficial biopsy, but the disease progression led to multiple organ failure, and the patient was considered too unstable to attempt the procedure. For AL patients without chemotherapy or stem cell transplant, survival is often one year from the time of diagnosis, but can range from three to twelve months depending on the degree of cardiac dysfunction [6]. With limited hospital records, we were unable to determine the onset of the patient’s symptoms or whether previous visits at other institutions indicated workup to diagnose amyloidosis.

## 4. Conclusions

A positive fat pad biopsy or FNA helps diagnose amyloidosis, but the procedure’s variable sensitivity misses many AL cases. This case report reinforces that a negative result does not exclude systemic AL with multisystem involvement and several fat pad biopsies can be negative in cases with high disease burden. In cases of possible multiple myeloma or MGUS, a bone marrow biopsy may be the preferred superficial biopsy. Although the procedure cannot be performed at the bedside, a rectal biopsy could be a prudent option given its higher sensitivity compared to fat pad biopsy. If the facility has the equipment and expertise, an involved organ biopsy may assist more in early diagnosis and guide appropriate treatment based on subtype. Institutional resources, technical expertise, a patient’s condition, and clinical suspicion all should be considered when deciding which biopsy method to pursue.

## Data Availability

No new data were created or analyzed in this study. Data sharing is not applicable to this article.
